# AC005034.3/hsa-miR-126-5p/EIF3H axis: bioinformatics analysis, expression validation, and association with prognosis and immunosuppressive microenvironment in pancreatic adenocarcinoma

**DOI:** 10.3389/fcell.2026.1725060

**Published:** 2026-02-25

**Authors:** Kai Sun, Zhi-xin Song, Xiao-yun Zhang, Xue-xing Wang, Song Wen, Ke-run Wang, Yi-lian Qiu

**Affiliations:** 1 Department of Oncology, The Affiliated Cancer Hospital of Gannan Medical University (Ganzhou Cancer Hospital), Ganzhou, Jiangxi, China; 2 Department of Pathology, Zhangye People’s Hospital Affiliated to Hexi University, Zhangye, Gansu, China

**Keywords:** EIF3H, immune infiltration, ncRNAs, pancreatic adenocarcinoma, prognosis

## Abstract

**Background:**

Pancreatic adenocarcinoma (PAAD) ranks among the most lethal human solid tumors, distinguished by its swift progression and limited effective treatment options. The eukaryotic translation initiation factor 3 subunit H (EIF3H) is postulated to be a critical factor in translational initiation, with emerging research indicating its potential involvement in promoting tumor invasion and metastasis. Nevertheless, the precise role of EIF3H within tumors remains insufficiently understood.

**Methods and Results:**

Employing a comprehensive pan-cancer methodology, we conducted an analysis of datasets from The Cancer Genome Atlas (TCGA), Gene Expression Omnibus (GEO), ArrayExpress, and the International Cancer Genome Consortium (ICGC). This analysis systematically assessed the prognostic significance, clinical associations, signaling pathways, immune infiltration profiles, and chemotherapeutic sensitivity associated with EIF3H expression. Through a series of correlation, expression, and survival analyses, we identified noncoding RNAs (ncRNAs) that contribute to the upregulation of EIF3H in pancreatic adenocarcinoma (PAAD). Notably, we identified the AC005034.3/hsa-miR-126-5p axis as the most promising upstream ncRNA-related pathway influencing EIF3H expression in PAAD. The differential expression of these three genes between PAAD and normal pancreatic tissues was further validated using reverse transcription-quantitative polymerase chain reaction (RT-qPCR) and immunohistochemistry (IHC). Additionally, we demonstrated significant correlations between EIF3H expression and immune infiltration profiles, as well as chemotherapeutic sensitivity in PAAD. Furthermore, we constructed a protein-protein interaction (PPI) network and performed functional annotations involving EIF3H.

**Conclusion:**

Our findings indicate that ncRNA-driven overexpression of EIF3H is associated with poor prognosis and tumor immune cell infiltration in PAAD, suggesting that the AC005034.3/hsa-miR-126-5p/EIF3H axis may serve as a promising prognostic biomarker and therapeutic target in PAAD.

## Introduction

Pancreatic adenocarcinoma (PAAD) remains one of the most challenging malignancies to treat, primarily due to its aggressive nature and late-stage diagnosis (Wood et al., 2022). Traditional treatments, such as surgery, radiation therapy, chemotherapeutics and endocrine therapy have shown limited success in patients with PAAD (2). Recent advancements in understanding the molecular and genomic landscape of PAAD have opened new avenues for targeted therapies and personalized medicine (Brown and Blair, 2025; Fiorillo et al., 2025). One significant development is the identification of KRAS mutations, which are prevalent in nearly all PAAD cases and serve as a critical driver of the disease (Varghese et al., 2025). The clinicogenomic landscape of PAAD highlights the prognostic potential of KRAS mutant allele dosage, which correlates with disease progression and overall survival, thereby offering a potential biomarker for therapeutic targeting (Pushalkar et al., 2018; Bednar and Pasca di Magliano, 2020; Fujiwara et al., 2020; Yamamoto et al., 2020).

The exploration of novel therapeutic agents continues to be a focal point in PAAD research (Wu et al., 2025a). The phase I study of AMG 193, a PRMT5 inhibitor, demonstrated encouraging antitumor activity in MTAP-deleted tumors, including PAAD, highlighting the potential of targeting specific genetic alterations for therapeutic gain (Rodon et al., 2024). Similarly, the development of mesothelin-specific nanobody conjugates represents a promising targeted therapy approach, leveraging the overexpression of mesothelin in PAAD to enhance drug delivery and efficacy (Yi et al., 2025). Moreover, the role of oxidative stress and redox signaling in PAAD pathogenesis is gaining attention. The interplay between oxidative stress and mutant KRAS is crucial in the initiation and progression of PAAD, suggesting that targeting redox pathways could complement existing therapeutic strategies (Sastre et al., 2025). This is further supported by findings that highlight the potential of RAS-GTP inhibitors, such as RMC-6236, which have shown promising results in preclinical models and early-phase clinical trials for RAS-driven cancers, including PAAD (Jiang et al., 2024). In summary, combining genomic insights, new therapies, and advanced knowledge of the tumor microenvironment and redox biology is leading to more effective, personalized treatments for pancreatic adenocarcinoma. Ongoing research and clinical trials are crucial for enhancing patient outcomes.

The eukaryotic translation initiation factor 3 (EIF3) is a substantial multiprotein complex, initially comprising five core subunits in budding yeast, while in mammals, it consists of 12 core subunits (Gomes-Duarte et al., 2018; Mestre-Fos et al., 2025). EIF3 represents the largest of the initiation factors that associates with the 40S ribosomal subunit, thereby preventing its premature association with the 60S subunit (Hashem et al., 2013; Choe et al., 2018). EIF3 plays a pivotal role throughout the translation pathway, particularly during all stages of translation initiation (des Georges et al., 2015; Zeman et al., 2019). In addition to its fundamental role in translation initiation, EIF3 has been demonstrated to: (Wood et al., 2022): facilitate reinitiation on downstream cistrons by remaining associated with early elongating ribosomes; (Halbrook et al., 2023); enhance programmed stop codon readthrough; and (Brown and Blair, 2025) promote ribosomal recycling and regulate translation termination *in vitro* (Syriste et al., 2024). However, the precise mechanisms by which EIF3 contributes to these processes remain unclear. EIF3 is composed of at least 13 subunits, designated EIF3A through EIF3M (Ramat et al., 2020; Sun et al., 2013). In mammals, EIF3 includes six subunits (EIF3A, EIF3B, EIF3C, EIF3E, EIF3F, and EIF3H). Among these, EIF3A, EIF3B, and EIF3C are evolutionarily conserved, whereas EIF3E, EIF3F, and EIF3H are not conserved through evolution (Sun et al., 2013; Meleppattu et al., 2015; Lin et al., 2020).

Eukaryotic translation initiation factor 3 subunit H (EIF3H) is a component of the EIF3 complex, encoded by the genes EIF3HA and EIF3HB in zebrafish (Choe et al., 2018; Cappuzzo et al., 2009; Jin et al., 2024). During protein translation, EIF3H plays a regulatory role in the initiation and elongation phases, as well as in modulating cellular growth rates (Zhou et al., 2020). Numerous studies have demonstrated that EIF3H is frequently amplified and overexpressed in various cancers (Chattopadhyay et al., 2025; Gao et al., 2025; Zhang et al., 2025a). Moreover, the amplification of the EIF3H gene has been associated with prostate cancer metastasis and poor prognosis, indicating that EIF3H may play a functional role in the proliferation of cancer cells (Pittman et al., 2010; Hu et al., 2020). Some reports suggest that EIF3H facilitates cancer cell growth by enhancing protein synthesis (Simon et al., 2016; Cappuzzo et al., 2009; Zhou et al., 2020; Poncová et al., 2019).

Recent investigations have revealed that within the Hippo-YAP signaling pathways, EIF3H regulates protein translation and influences the stability of Yes-associated protein-1 (YAP1), thereby promoting breast tumor metastasis. Nonetheless, the precise roles and functions of EIF3H in human tumors remain inadequately understood and warrant further elucidation. In our previous study, we demonstrated a correlation between YAP1 and patient outcomes, as well as tumor immune cell infiltration, in pancreatic cancer (Sun et al., 2021). Consequently, in the present study, we aimed to investigate the role and underlying mechanisms of EIF3H in PAAD, while also assessing its expression pattern and prognostic significance across various cancer types. Employing a comprehensive pan-cancer methodology, as emphasized by recent studies on multi-cohort validation for robust biomarkers (Ma et al., 2024; Chen et al., 2025), we conducted an analysis of datasets from TCGA, GEO, ArrayExpress, and ICGC. Subsequently, we identified non-coding RNAs (ncRNAs) associated with the elevated expression of EIF3H in PAAD, and we highlighted the AC005034.3/hsa-miR-126-5p axis as a key regulatory pathway in this context. To validate these findings, we confirmed the differential expression of these genes in PAAD tissues compared to normal tissues using RT-qPCR and IHC. Furthermore, we discovered significant correlations between EIF3H expression and immune cell infiltrates as well as immune checkpoints in PAAD. Additionally, we constructed protein–protein interaction (PPI) networks and performed functional annotations for EIF3H, which suggested that ncRNA-driven upregulation of EIF3H was associated with poor prognosis and tumor immune cell infiltration in PAAD.

## Materials and methods

### Data processing and differential expression, survival and correlation analysis

Our study utilized RNA-seq raw count data along with matched clinical metadata from both tumor and adjacent normal tissues across 33 cancer types, sourced from TCGA and Genotype-Tissue Expression (GTEx) databases. Specifically, for PAAD, we analyzed 179 tumor samples, 4 adjacent normal tissues, and 167 healthy pancreatic specimens. Detailed clinicopathological characteristics of TCGA-PAAD are presented in Supplementary Table S1. To enhance the robustness of our findings, we integrated additional PAAD datasets from the Gene Expression Omnibus (GEO) platform, comprising: GSE28735 (45 tumor and 45 adjacent normal tissues), GSE57495 (63 PAAD cases), GSE62452 (69 PAAD and 61 adjacent normal tissues), GSE71729 (145 primary and 61 metastatic tumors, 17 cell lines, 46 pancreatic and 88 distant site normal tissues), GSE78229 (50 PAAD samples), and GSE79668 (51 PAAD cases) (Barrett et al., 2013). Further datasets were acquired from the International Cancer Genome Consortium (ICGC) portal and the E_MTAB_6134 repository (309 PAAD samples) available through ArrayExpress. Gene expression levels were normalized using transcripts per million (TPM) values followed by log2 (TPM+1) transformation. All computational procedures were executed in R version 4.3.0, with detailed documentation to maintain alignment with contemporary cancer bioinformatics protocols. Data imputation for missing values was performed using the missForest package (Stekhoven and Bühlmann, 2012). Statistical analyses were conducted with R version 3.6.3, employing the “ggplot2”, “survminer” and “survival” packages for visualization of expression patterns and survival outcomes (Ito and Murphy, 2013). Statistical significance was evaluated through log-rank testing, while univariate Cox regression models provided hazard ratios (HR) with corresponding 95% confidence intervals (CI) and p-values. Gene-gene interactions were examined using the “ggstatsplot” package, with quantitative variable associations assessed via Pearson or Spearman correlation coefficients as appropriate.

### Tissue specimen collection and immunohistochemical analysis

The investigation employed 12 paired specimens comprising PAAD and corresponding adjacent normal pancreas tissues collected from Ganzhou Cancer Hospital. Ethical approval for this study was granted by the Institutional Review Board (Ethics Approval Code: 2025Kelunshen101). All tissue samples were subjected to thorough histopathological examination to validate PAAD diagnosis, with detailed clinicopathological characteristics documented in Supplementary Table S2. The inclusion criteria specified: (Wood et al., 2022): histologically confirmed PAAD cases, (Halbrook et al., 2023), availability of complete clinical records. Exclusion parameters included: (Wood et al., 2022): inconclusive pathological findings, (Halbrook et al., 2023), incomplete clinical data, (Brown and Blair, 2025), history of significant systemic therapy. For immunohistochemical analysis, specimens were preserved in 10% neutral buffered formalin, embedded in paraffin, and sectioned at 4 μm thickness. Following standard deparaffinization and rehydration procedures, antigen retrieval was conducted using citrate buffer solution (1:100 dilution; Boster Biological Technology, China). Tissue sections were subsequently incubated with HRP-conjugated secondary antibodies (ZSGB-Bio, China), developed with DAB chromogen, and counterstained with hematoxylin. Quantitative assessment of staining intensity was performed using Image-Pro Plus 6.0 software (Media Cybernetics, United States), with integrated optical density measurements obtained from multiple high-power fields for each specimen.

### Prognostic significance of EIF3H in PAAD

To evaluate patient outcomes, we conducted detailed survival assessments using the Kaplan–Meier method, which allowed for the comparison of major clinical endpoints, including overall survival (OS), progression-free survival (PFS), disease-free survival (DFS), and disease-specific survival (DSS), between patient groups stratified by high and low PAAD expression levels, defined according to median expression values. These analyses were implemented with the survival package (version 3.3-1). Differences in survival outcomes were assessed for statistical significance using the log-rank test, with a significance level set at P < 0.05. Survival probability curves, complete with 95% confidence intervals and median survival durations for each subgroup, were generated using the survminer package (version 0.4.9). To further evaluate prognostic performance, time-dependent receiver operating characteristic (ROC) analysis was carried out with the “timeROC” package, estimating survival probabilities at 1, 3, and 5 years, generating associated ROC curves, and calculating area under the curve (AUC) values (Chen and Wang, 2020). External validation of EIF3H expression patterns in PAAD was performed using independent datasets sourced from GEO and ICGC to enhance reliability (Barrett et al., 2013; Chandrashekar et al., 2017). Additionally, both univariate and multivariate Cox proportional hazards regression models were employed to systematically assess potential prognostic variables.

### GEPIA2 database analysis

Gene Expression Profiling Interactive Analysis 2 (GEPIA2) is used to analyze standard processing pipelines of RNA sequencing expression data in 9,736 tumors and 8,587 normal samples from TCGA and GTEx projects (Tang et al., 2019). We used GEPIA2 to explore EIF3H mRNA expression levels in different cancers specimens and that in normal specimens, we also used GEPIA2 to assess the associations between EIF3H mRNA expression and patient prognosis in pan-cancers. Then We examined the relationship of the expression levels of hsa-miR-126-5p and AC005034.3 in PAAD, HNSC and their corresponding adjacent tissues, and the associations of hsa-miR-126-5p and AC005034.3 expression levels and PAAD, HNSC patient’s prognosis.

### Kaplan-Meier plotter analysis

Kaplan-Meier plotter (KM plotter; http://kmplot.com/analysis/),acting as an online database, is used to explore the influence of multiple genes on the prognosis of 21 different types of cancers. KM plotter contains microarray gene expression data and survival information via TCGA, European Genome-Phenome Archive and Gene Expression Omnibus (Nagy et al., 2021). We used KM plotter to explore the prognostic values of EIF3H, hsa-miR-126-5p and AC005034.3 in pan-cancers.

### Candidate miRNA prediction

We predicted Upstream binding miRNAs of EIF3H via several target gene prediction website, consisting of TargetScan (Agarwal et al., 2015), miRDB (Ramat et al., 2020; Sun et al., 2013),miRcode, miRmap (Vejnar and Zdobnov, 2012),miRWalk (Sticht et al., 2018), DIANA-microT (Paraskevopoulou et al., 2013) and ENCORI (Li et al., 2014). We picked up candidate miRNAs that commonly appeared more than three programs as mentioned above for subsequent analyses. These predicted miRNAs were chosen as candidate miRNAs of EIF3H.

### ENCORI database analysis

The Encyclopedia of RNA Interactomes (ENCORI) database is a database for discovering the connection between miRNA-ncRNA, miRNA-mRNA, ncRNA-RNA, RNA-RNA, RBP-mRNA and RBP-ncRNA (Ramat et al., 2020; Sun et al., 2013). We used ENCORI to predict upstream potential miRNAs and lncRNAs that interact with EIF3H and hsa-miR-126-5p.

### The Association Between EIF3H Expression and Immune Cell Infiltration in PAAD

To investigate the relationship between EIF3H expression and tumor microenvironment characteristics in PAAD, we performed an integrative immunogenomic assessment utilizing multi-omics datasets obtained from TCGA, GEO, ICGC, and ArrayExpress repositories. Our analytical strategy employed seven advanced computational approaches for immune cell characterization (ssGSEA, xCell, CIBERSORT, EPIC, TIMER, MCP-counter, and quanTIseq) implemented via specialized R packages such as immunedeconv, estimate, and GSVA. This comprehensive methodology facilitated detailed examination of immune cell infiltration patterns, stromal components, immune activation markers, and genomic instability features including tumor mutational burden (TMB) and microsatellite instability (MSI). Additionally, we analyzed the correlation between EIF3H expression and 150 immune-related genes categorized into five functional groups: (Wood et al., 2022): chemokine signaling pathways (41 genes), (Halbrook et al., 2023), immune checkpoint molecules (18 genes), (Brown and Blair, 2025), antigen processing and presentation components (21 genes), (Fiorillo et al., 2025), immunosuppressive factors (24 genes), and (Varghese et al., 2025) immunostimulatory molecules (46 genes) (Ito and Murphy, 2013; Sturm et al., 2020; Gu, 2022). All computational procedures were executed using R statistical software (version 4.3.0), with data visualization performed using ggplot2, pheatmap, and ggstatsplot packages to ensure accurate statistical analysis and clear graphical presentation of findings.

### Drug sensitivity of EIF3H in PAAD

Drug sensitivity data were systematically collected from three authoritative public repositories: the Cancer Therapeutics Response Portal (CTRP version 2.0), the PRISM drug repurposing collection, and the Genomics of Drug Sensitivity in Cancer (GDSC) platform. To examine possible associations between EIF3H expression levels and pharmacological responses, we conducted Spearman correlation analyses evaluating 217 therapeutic agents, including kinase-targeted drugs, epigenetic modulators, and conventional chemotherapeutic compounds. All statistical computations were performed using R software (version 4.3.0), employing the tidyverse collection for data processing, the pRRophetic tool for drug response prediction modeling, and ComplexHeatmap for detailed visualization of analytical results (Gu, 2022).

### Protein–protein interaction (PPI) networks and functional enrichment analysis

STRING is an online database designed for evaluating interacting genes (Szklarczyk et al., 2019). In this study, we used STRING to search co-expression genes of EIF3H and construct PPI networks. We used R package “ClusterProfiler” to perform Gene ontology (GO) enrichment and Kyoto Encyclopedia of Genes and Genomes (KEGG) pathway analyses of co-expression genes, and visualized by R package “ggplot2”.

### Cell lines and cell culture

The human pancreatic ductal epithelial (HPDE) cell line and pancreatic ductal adenocarcinoma (PANC-1) cell line were obtained from iCell Bioscience Inc. (Shanghai, China). For cell culture and maintenance, HPDE cells were cultured in Dulbecco’s Modified Eagle Medium (DMEM; Procell) supplemented with 10% fetal bovine serum (FBS; Procell). PANC-1 cells, by contrast, were cultured in Roswell Park Memorial Institute 1,640 medium (RPMI-1640; Procell) also supplemented with 10% FBS (Procell). Both cell lines were maintained in a humidified incubator under standard conditions (37 °C, 5% CO_2_).

### RNA extraction and RT-qPCR assay

Relative transcript levels of AC005034.3, hsa-miR-126-5p, and EIF3H were quantified via reverse transcription quantitative polymerase chain reaction (RT-qPCR). Total RNA was extracted using TRIzol reagent (Vazyme Biotech) following standard protocols. First-strand cDNA synthesis was performed with 5× HiScript qRT SuperMix II (YEASEN) in accordance with the manufacturer’s instructions. Quantitative amplification was conducted on an Mx3000P QPCR system (Thermo Fisher Scientific, CA) utilizing ChamQ Universal SYBR qPCR Master Mix (YEASEN). Thermocycling parameters were as follows: initial denaturation at 95 °C for 10 s, followed by 40 amplification cycles comprising denaturation at 95 °C for 15 s and combined annealing/extension at 60 °C for 60 s.

Expression levels were determined using the 2^−ΔΔCT^ method, with β-actin mRNA serving as the endogenous reference gene for normalization. Technical triplicates were included for each sample to ensure experimental reproducibility. Oligonucleotide primer sequences specific to target genes are provided below:

hsa-miR-126-5p-F: CCG​CCA​TTA​TTA​CTT​TTG​GTA​CGC​G. (The reverse primer for hsa-miR-126-5p is the kit-supplied primer of Sangon Biotech’s tailing-based reverse transcription kit.)

H-AC005034.3-F: GGT​TCT​GAC​AAC​TGT​AAC​ACC​TA.

H-AC005034.3-R: CTG​TCC​TCT​GGA​CTC​ATC​ATT​G.

H-EIF3H-F: ACT​CCT​GGA​CTC​TCA​GTT​TAG​T.

H-EIF3H-R: GGA​GTC​AGT​CTG​TAT​GCC​TTT​AG.

H-β-actin-F: GGA​AAT​CGT​GCG​TGA​CAT​TAA​G.

H-β-actin-R: AGC​TCG​TAG​CTC​TTC​TCC​A.

### Statistical analysis

Statistical analyses were conducted using R (version 4.3.0), employing a range of methods to thoroughly evaluate the dataset. Quantitative evaluations included the calculation of fold-change values and hazard ratios (HR), with statistical significance assessed via Log-rank tests. Associations between variables were examined using both Spearman’s and Pearson’s correlation coefficients. Comparative analyses across groups were performed using Wilcoxon rank-sum tests, Student’s t-tests (for two-group comparisons), and ANOVA (for comparisons involving more than two groups). Survival outcomes were graphically represented using Kaplan–Meier curves, and between-group differences were tested with log-rank tests, applying a significance threshold of *P* < 0.05. Statistical significance was graphically represented using asterisks: * (*P* < 0.05), ** (*P* < 0.01), *** (*P* < 0.001) and **** (*P* < 0.0001).

## Results

### Assessment of EIF3H expression in different cancers and normal tissues

**FIGURE 1:**
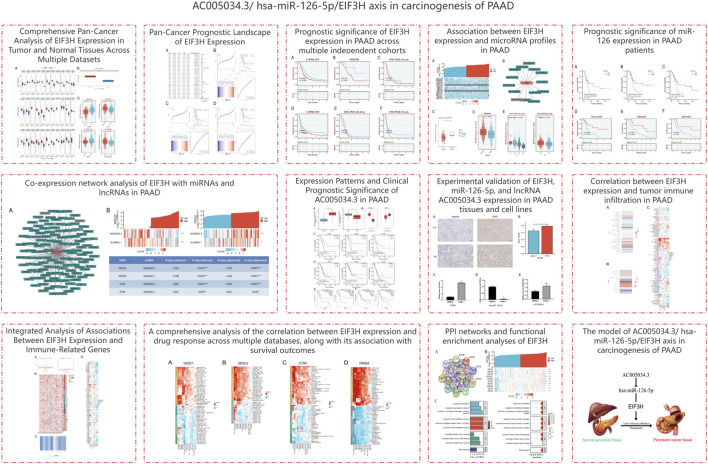
The research flowchart of this study.

**FIGURE 2:**
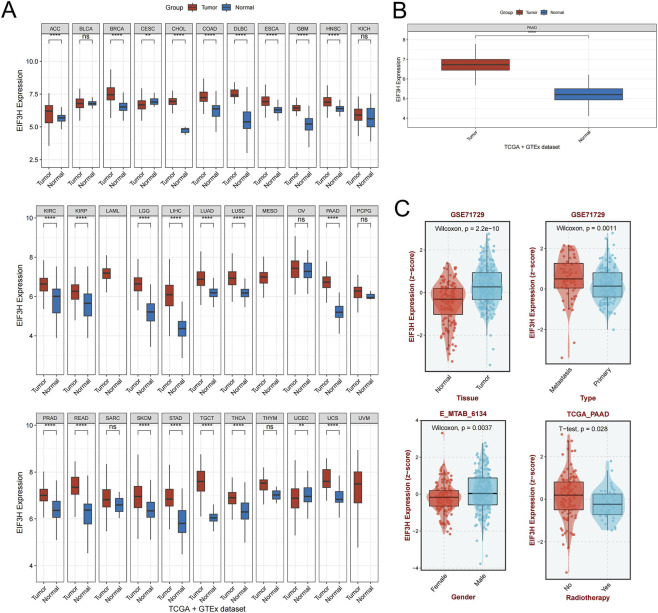
Comprehensive Pan-Cancer Analysis of EIF3H Expression in Tumor and Normal Tissues Across Multiple Datasets. **(A)** Differential EIF3H expression between tumor and normal tissues across 33 cancer types from TCGA and GTEx databases. **(B)** Combined analysis of EIF3H expression patterns in tumor versus normal tissues from integrated TCGA and GTEx datasets. **(C)** Validation of EIF3H expression differences in independent GEO datasets (GSE71729, etc.). *P < 0.05, **P < 0.01, ***P < 0.001, ****P < 0.0001. Abbreviations: EIF3H, eukaryotic translation initiation factor 3 subunit H; PAAD, Pancreatic adenocarcinoma; TCGA, The Cancer Genome Atlas.

The study design flowchart is depicted in Figure 1. We explored the mRNA expression levels of EIF3H in tumor tissues and adjacent tissues from 33 types of cancer via TCGA dataset and GTEX databases. Our research presented that the mRNA expression levels of EIF3H were higher than adjacent and normal tissues control in most tumor types, such as Adrenocortical carcinoma (ACC), Breast invasive carcinoma (BRCA), Cholangio carcinoma (CHOL), Colon adenocarcinoma (COAD), Lymphoid neoplasm diffuse large b-cell lymphoma (DLBC), Esophageal carcinoma (ESCA), Glioblastoma multiforme (GBM), Head and Neck squamous cell carcinoma (HNSC), Kidney renal clear cell carcinoma (KIRC), Kidney renal papillary cell carcinoma (KIRP), Brain Lower Grade Glioma (LGG), Liver hepatocellular carcinoma (LIHC), Lung adenocarcinoma (LUAD), Lung squamous cell carcinoma (LUSC), Pancreatic adenocarcinoma (PAAD), prostate adenocarcinoma (PRAD), Rectum adenocarcinoma (READ), Skin Cutaneous Melanoma (SKCM), stomach adenocarcinoma (STAD), Testicular germ cell tumors (TGCT), thyroid Cancer (THCA), and Uterine Carcinosarcoma (UCS) (Figure 2A). On the contrary, EIF3H mRNA expression levels were lower in Cervical squamous cell carcinoma and endocervical adenocarcinoma (CESC) and Uterine Corpus Endometrial Carcinoma (UCEC). The distinct expression signature of EIF3H underscores its tissue-specific functional implications in diverse malignancies, providing a framework for future mechanistic studies. Emerging evidence indicates that EIF3H may exert tumor-suppressive effects in CESC and UCEC, warranting further investigation into its cancer subtype-specific regulatory functions. Our analysis extended to PAAD expression dynamics in PAAD, where TCGA-PAAD data demonstrated marked upregulation in tumor versus normal adjacent tissues, a finding corroborated in the GSE71729 dataset (Figures 2B,C). In PAAD, the expression of EIF3H was found to be significantly associated with the response to radiotherapy (*p* = 0.028), with cases responsive to treatment exhibiting elevated expression levels. Analysis of the E_MTAB_6134 cohort revealed a marked gender dimorphism, with male patients showing higher EIF3H expression compared to female patients. Furthermore, data from the GSE71729 dataset indicated that metastatic lesions exhibited significantly higher EIF3H expression than primary tumors, suggesting a potential role in tumor progression (Figure 2C).

### Correlations between expression of EIF3H and prognosis of pan-cancer patients

**FIGURE 3:**
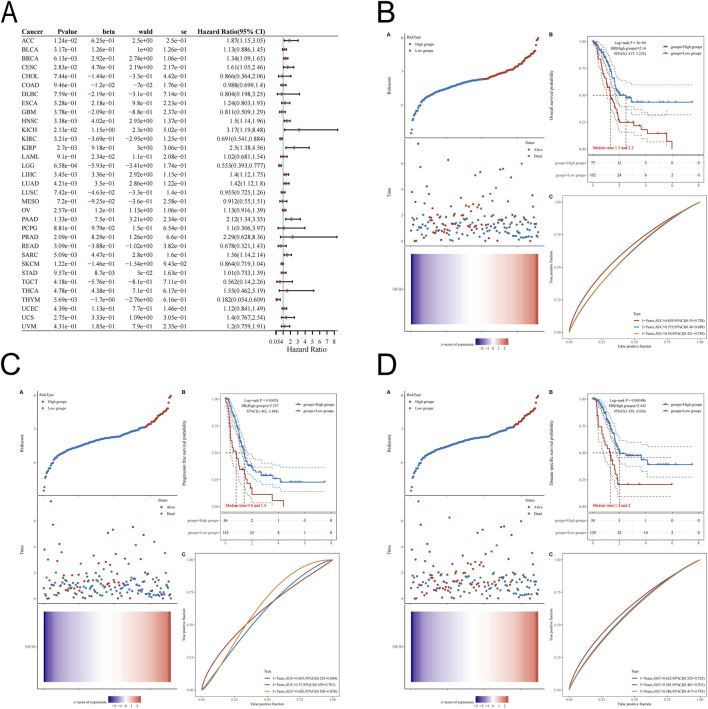
Pan-Cancer Prognostic Landscape of EIF3H Expression. **(A)** Prognostic associations between EIF3H expression and overall survival across 33 cancer types from TCGA database. **(B)** Prognostic analysis for OS of EIF3H in the TCGA-KIRC dataset. **(C)** Prognostic analysis for PFS of EIF3H in the TCGA-KIRC dataset. **(D)** Prognostic analysis for DSS of EIF3H in the TCGA-KIRC dataset. Abbreviations: EIF3H, eukaryotic translation initiation factor 3 subunit H; PAAD, Pancreatic adenocarcinoma; TCGA, The Cancer Genome Atlas; GEO, Gene Expression Omnibus.

**FIGURE 4:**
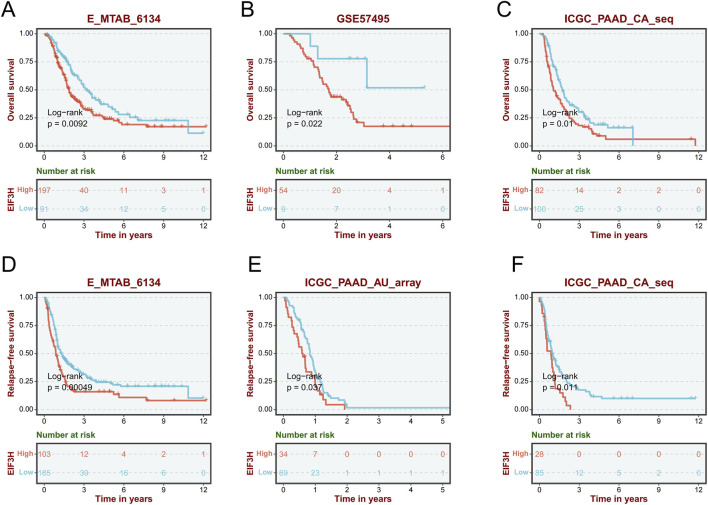
Prognostic significance of EIF3H expression in PAAD across multiple independent cohorts. **(A)** Kaplan–Meier OS analysis of PAAD patients stratified by EIF3H expression in the E-MTAB-6134 cohort. **(B)** OS analysis in the GSE57495 cohort. **(C)** OS analysis in the ICGC_PAAD_CA cohort. **(D)** RFS analysis in the E-MTAB-6134 cohort. **(E)** RFS analysis in the GSE57495 cohort. **(F)** RFS analysis in the ICGC_PAAD_CA cohort. *P < 0.05, **P < 0.01, ***P < 0.001. Abbreviations: OS, overall survival; RFS, relapse-free survival. DSS, disease-specific survival.

The prognostic significance of EIF3H expression across multiple cancer types using univariate Cox regression analysis. Elevated EIF3H expression was significantly associated with worse overall survival in several malignancies, including ACC (HR = 1.87, *p* = 0.0124), BRCA (HR = 1.34, 95% CI: 1.09–1.65, *p* = 0.00613), CESC (HR = 1.61, *p* = 0.0283), HNSC (HR = 1.50, *p* = 0.00338), KICH (HR = 3.17, 95%, *p* = 0.0213), KIRP (HR = 2.50, *p* = 0.0027), LIHC (HR = 1.40, *p* = 0.00345), LUAD (HR = 1.42, *p* = 0.00421), SARC (HR = 1.56, p = 0.00509) and PAAD (HR = 2.12, *p* = 0.00133), among others. Conversely, it was a favorable prognostic factor in KIRC (HR = 0.691, p = 0.00321), LGG (HR = 0.553, p = 0.000658), and THYM (HR = 0.182, p = 0.00569) (Figure 3A). Further Kaplan-Meier analysis in the TCGA PAAD cohort confirmed that high EIF3H expression was significantly associated with poorer overall survival (HR = 2.14), progression-free survival (PFS, HR = 2.257), and disease-specific survival (DSS, HR = 2.443) (Figures 3B–D). Time-dependent ROC analysis demonstrated that EIF3H expression possessed predictive capability for 1-, 3-, and 5-year survival, with AUC values ranging from 0.575 to 0.682. These findings strongly suggested that EIF3H functioned as a significant prognostic biomarker in a pan-cancer context, particularly in PAAD. To evaluate the prognostic significance of EIF3H in PAAD, KM survival analyses were conducted across multiple independent cohorts (Figure 4). The analyses illustrated OS curves stratified by EIF3H expression in the E_MTAB_6134 (log-rank *p* = 0.0092), GSE57495 (log-rank *p* = 0.022), and ICGC_PAAD_CA_seq (log-rank p = 0.01) datasets, respectively (Figures 4A–C). In these datasets, patients with high EIF3H expression exhibited significantly shorter OS compared to those with low expression. Similarly, RFS curves were depicted in the E_MTAB_6134 (log-rank *p* = 0.0049), ICGC_PAAD_AU_array (log-rank *p* = 0.037), and ICGC_PAAD_CA_seq (log-rank *p* = 0.011) datasets, demonstrating that high EIF3H expression was also associated with poorer RFS (Figures 4D–F). The consistent findings across multiple independent datasets reinforced the evidence that EIF3H served as a robust and reliable prognostic biomarker in PAAD.

### Analysis of upstream miRNAs of EIF3H

**FIGURE 5:**
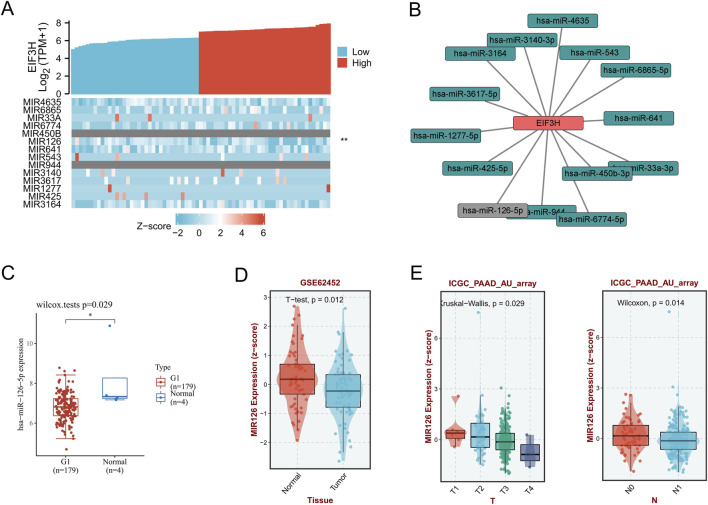
Association between EIF3H expression and microRNA profiles in PAAD. **(A)** Heatmap showing co-expression patterns of EIF3H and selected miRNAs in PAAD. **(B)** miRNA-EIF3H regulatory network. **(C)** Differential expression of hsa-miR-126-5p between PAAD tumor samples (G1, n = 179) and normal tissues (n = 4). **(D)** Validation of miR-126 expression in GSE62452 comparing tumor and normal tissues. **(E)** Correlation between miR-126 expression and lymph node metastasis and tumor stage (T1–T4) in ICGC_PAAD_AU_array. Abbreviations: PAAD, pancreatic adenocarcinoma; miRNA, microRNA.

Increasing evidence that ncRNAs regulate gene expression by various manners at almost every step (Li et al., 2018; Park et al., 2018). LncRNAs affects miRNA affinity of target mRNA by attaching to the similar miRNA response elements, thereby regulating gene expression at the transcriptional level (Ahn et al., 2020; Zhang et al., 2021).To unravel whether EIF3H was regulated by some ncRNAs, we predicted upstream binding miRNAs of EIF3H via several target gene prediction website, TargetScan (Agarwal et al., 2015), miRDB (Ramat et al., 2020; Sun et al., 2013), miRcode, miRmap (Vejnar and Zdobnov, 2012), miRWalk (Sticht et al., 2018), DIANA-microT (Ramat et al., 2020; Sun et al., 2013) and ENCORI (Ramat et al., 2020; Sun et al., 2013), finally found 14 miRNAs (Figure 5A). Cystoscope software was used to establish a miRNA-EIF3H regulatory network. There should be a negative relationship between EIF3H and upstream miRNA in because of the mechanism that upstream miRNAs were negatively regulated the expression of EIF3H at the post-transcriptional level. So, the correlation between EIF3H and 14 miRNAs was detected via in PAAD TCGA database. As a result, our study showed that the expression levels of EIF3H were significance negatively associated with hsa-miR-126-5p (MIR126) (P < 0.01), and no statistical expression relationships between EIF3H and other miRNAs in PAAD (Figures 5A,B; Table 1). We quantified the mRNA expression levels of hsa-miR-126-5p in 179 PAAD tumor tissues and 4 adjacent non-tumor tissues by integrating data from TCGA and GTEx databases. Compared with adjacent non-tumor tissues, hsa-miR-126-5p expression was significantly downregulated in PAAD tumor tissues (Figure 5C). This finding was further validated in the GSE62452 dataset, where tumor tissues exhibited markedly reduced MIR126 expression relative to normal tissues (p < 0.0001). Moreover, in the ICGC_PAAD_AU_array dataset, MIR126 expression showed progressive downregulation with advancing tumor stage (T1 to T4) and was also decreased in tissues with N1 lymph node metastasis compared to those with N0 status. Collectively, these results indicated that the EIF3H/miR-126-5p axis functions as a clinically relevant regulatory pathway in pancreatic cancer progression (Figure 5D).

**TABLE 1:** Relationship between of EIF3H expression and upstream miRNAs expression in PAAD.

GENE	miRNA	R-value (Pearson)	P-value (Pearson)	R-value (Spearman)	P-value (Spearman)
EIF3H	hsa-miR-4635	0.137	0.255	0.130	0.279
EIF3H	hsa-miR-6865-5p	0.194	0.106	0.116	0.335
EIF3H	hsa-miR-33a-3p	−0.042	0.727	−0.029	0.810
EIF3H	hsa-miR-6774-5p	0.238	0.046	0.186	0.120
EIF3H	hsa-miR-450b-3p	NA	NA	NA	NA
EIF3H	hsa-miR-126-5p	−0.353	0.003**	−0.306	0.009**
EIF3H	hsa-miR-641	0.149	0.216	0.133	0.269
EIF3H	hsa-miR-543	−0.099	0.414	−0.009	0.938
EIF3H	hsa-miR-944	NA	NA	NA	NA
EIF3H	hsa-miR-3140-3p	−0.039	0.746	−0.103	0.394
EIF3H	hsa-miR-3617-5p	0.023	0.850	0.030	0.804
EIF3H	hsa-miR-1277-5p	0.066	0.583	0.045	0.709
EIF3H	hsa-miR-425-5p	−0.189	0.114	−0.188	0.116
EIF3H	hsa-miR-3164	0.055	0.651	0.018	0.882

^*^ *P* < 0.05, **P < 0.01, ***P < 0.001. The symbol * denotes statistical significance at *P* < 0.05, ** denotes statistical significance at *P* < 0.01, and *** denotes statistical significance at *P* < 0.001, all indicating statistically meaningful differences between comparison groups.

**FIGURE 6:**
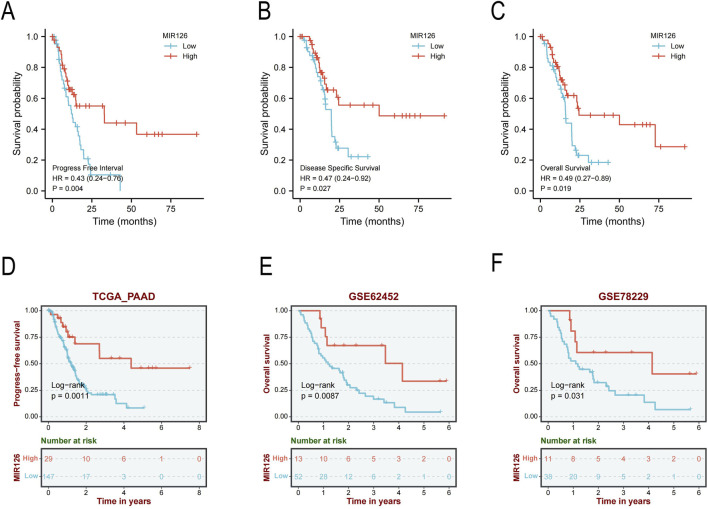
Prognostic significance of miR-126 expression in PAAD patients. **(A)** Progress-free interval analysis based on miR-126 expression levels. **(B)** Disease-specific survival analysis stratified by miR-126 expression. **(C)** OS analysis according to miR-126 expression status. **(D)** Validation of PFS in TCGA_PAAD cohort. **(E)** OS analysis in GSE62452 cohort. **(F)** OS analysis in GSE78229 cohort. p < 0.05, **p < 0.01, ***p < 0.001 Abbreviations: PAAD, pancreatic adenocarcinoma; HR, hazard ratio; CI, confidence interval; PFI, Progress-free interval; OS, overall survival; PFS, Progress-free survival. DSS, disease-specific survival.

We also explored the correlation between the expression levels of hsa-miR-126-5p and PAAD patient prognosis in TCGA and GEO database. In the TCGA-PAAD cohort, high MIR126 expression was associated with improved progression-free interval (PFI; HR = 0.43), DSS (HR = 0.47), OS (HR = 0.49) and PFS (Figures 6A–D). These findings were corroborated in the independent GEO cohorts (GSE62452 and GSE78229), where elevated MIR126 expression significantly correlated with better OS (Figures 6E,F). No significant heterogeneity was observed across cohorts, underscoring the robustness of MIR126 as a favorable prognostic biomarker in pancreatic cancer. Integrating correlation, expression, and survival analyses, we proposed that hsa-miR-126-5p may represent the most promising regulatory miRNA targeting EIF3H in PAAD.

### Analysis of upstream potential lncRNAs of hsa-miR-126-5p in PAAD

**FIGURE 7:**
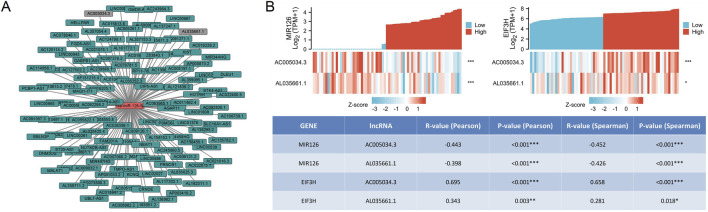
Co-expression network analysis of EIF3H with miRNAs and lncRNAs in PAAD. **(A)** lncRNA-hsa-miR-126-5p regulatory network; **(B)** Correlation analysis of hsa-miR-126-5p/EIF3H expression and AC005034.3 and AL035661.1 in PAAD in TCGA and GTEx database. *P < 0.05, **P < 0.01, ***P < 0.001.

**FIGURE 8:**
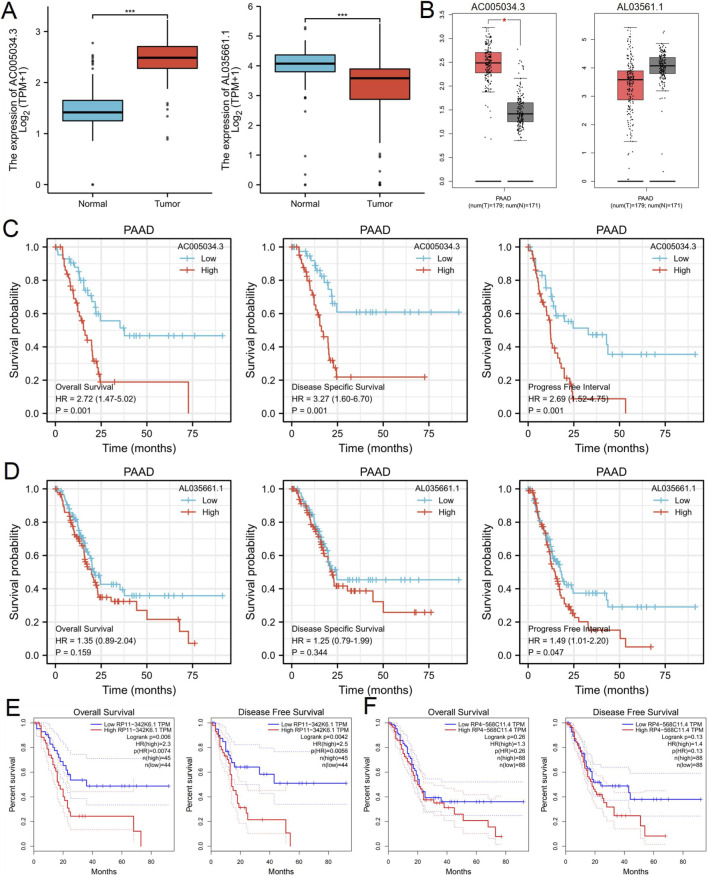
Expression Patterns and Clinical Prognostic Significance of AC005034.3 in PAAD. **(A)** AC005034.3 and AL035661.1 differentially expression levels in normal pancreatic tissues and PAAD tissues in TCGA and GTEx database; **(B)** AC005034.3 and AL035661.1 differentially expression levels in normal pancreatic tissues and PAAD tissues determined by GEPIA 2 database; **(C)** OS, DSS and PFI analysis for AC005034.3 in PAAD in TCGA database. **(D)** OS, DSS and PFI analysis for AL035661.1 in PAAD in TCGA database. **(E)** The OS and DFS analysis for AC005034.3 in PAAD determined by GEPIA 2 database in TCGA database; **(F)** The OS and DFS analysis for AL035661.1 in PAAD determined by GEPIA 2 database in TCGA database. *P < 0.05, **P < 0.01, ***P < 0.001 Abbreviations: OS, overall survival; DSS, disease-specific survival; PFI, progress free interval; DFS, disease-free survival.

We utilized the ENCORI database to predict potential upstream long non-coding RNAs (lncRNAs) that interact with hsa-miR-126-5p. Ultimately, we identified 124 candidate lncRNAs with potential interactions with hsa-miR-126-5p. The lncRNA-hsa-miR-126-5p regulatory network was visualized using Cytoscape software (Figure 7A). According to the competitive endogenous RNA (ceRNA) hypothesis, lncRNAs can competitively bind to tumor-suppressive microRNAs (miRNAs), thereby mitigating the suppressive effects of miRNAs on their target messenger RNAs (mRNAs). This hypothesis implies a negative correlation between lncRNAs and target miRNAs, alongside a positive correlation between lncRNAs and target mRNAs within the ceRNA network. Consequently, we evaluated the expression correlations between hsa-miR-126-5p/EIF3H and the 124 lncRNAs using data from the TCGA PAAD database. Our findings revealed that only AC005034.3 (lnc-POLE4-5) and AL035661.1 exhibited a positive association with EIF3H and a negative association with hsa-miR-126-5p (Figure 7B). Subsequently, we conducted an expression analysis of AC005034.3 and AL035661.1 in PAAD using the TCGA dataset. The analysis demonstrated that AC005034.3 expression levels were significantly upregulated in PAAD compared to normal controls (Figure 8A), whereas AL035661.1 exhibited the opposite trend (Figure 8A). Meanwhile we utilized the GEPIA database to confirm that the data were consistent with the previously mentioned results (Figure 8B). Subsequently, we assessed the prognostic significance of AC005034.3 and AL035661.1 in PAAD using the TCGA database. The findings indicated that elevated expression levels of AC005034.3 were significantly associated with poorer outcomes in PAAD, as evidenced by OS (HR = 2.72), DSS (HR = 3.27) and PFI (HR = 2.69) (Figure 8C). Conversely, no significant correlation was found between the expression levels of AL035661.1 and prognosis in PAAD patients (Figure 8D). Consistent with these findings, analysis using the GEPIA database also demonstrated that higher expression of AC005034.3 was significantly correlated with poorer prognosis (OS, HR = 2.3, P = 0.006; DFS, HR = 2.5, P = 0.0042), while no correlation was observed for AL035661.1 expression with prognosis in PAAD patients (Figures 8E,F). Based on correlation analysis, expression analysis, and survival analysis, AC005034.3 was identified as the most promising upstream lncRNA of the EIF3H/hsa-miR-126-5p axis in PAAD.

### Experimental validation of mRNA and protein expression of three genes in PAAD

**FIGURE 9:**
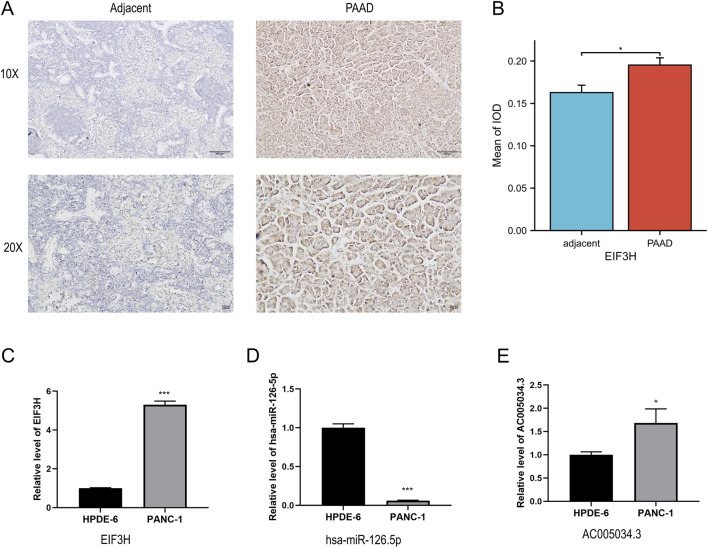
Experimental validation of EIF3H, miR-126-5p, and lncRNA AC005034.3 expression in PAAD tissues and cell lines. **(A)** Representative IHC staining of EIF3H in adjacent normal and PAAD tissues. **(B)** Quantitative analysis of EIF3H IOD in normal and PAAD tissues. **(C)** qRT-PCR analysis of EIF3H mRNA expression in normal pancreatic (HPDE-6) and PAAD (PANC-1) cell lines. **(D)** qRT-PCR analysis of miR-126-5p expression in HPDE-6 and PANC-1 cell lines. **(E)** qRT-PCR analysis of lncRNA AC005034.3 expression in HPDE-6 and PANC-1 cell lines. *P < 0.05, **P < 0.01, ***P < 0.001 Abbreviations: PAAD, pancreatic adenocarcinoma; IHC, immunohistochemistry; IOD, integrated optical density; qRT-PCR, quantitative reverse transcription polymerase chain reaction; lncRNA, long non-coding RNA.

Based on the experimental validation presented in Figures 9A,B, immunohistochemical analysis of clinical specimens revealed markedly stronger EIF3H staining in PAAD tissues compared to adjacent normal tissues, which was further quantified by a significant increase in the mean integrated optical density. At the cellular level, qRT-PCR analysis demonstrated that EIF3H mRNA expression was significantly upregulated in PANC-1 pancreatic cancer cells relative to normal HPDE-6 pancreatic ductal epithelial cells (Figure 9C). Conversely, hsa-miR-126-5p expression was substantially downregulated in the cancer cells, showing an inverse relationship with EIF3H expression (Figure 9D). Furthermore, the long non-coding RNA AC005034.3 exhibited significant overexpression in PANC-1 cells compared to HPDE-6 controls, suggesting its potential involvement in the EIF3H regulatory network in pancreatic carcinogenesis (Figure 9E). Collectively, these experimental results consistently validate our previous bioinformatic findings, confirming the dysregulated expression patterns of EIF3H, miR-126-5p, and AC005034.3 in PAAD.

### The expression of EIF3H associated with immune cell infiltration in PAAD

**FIGURE 10:**
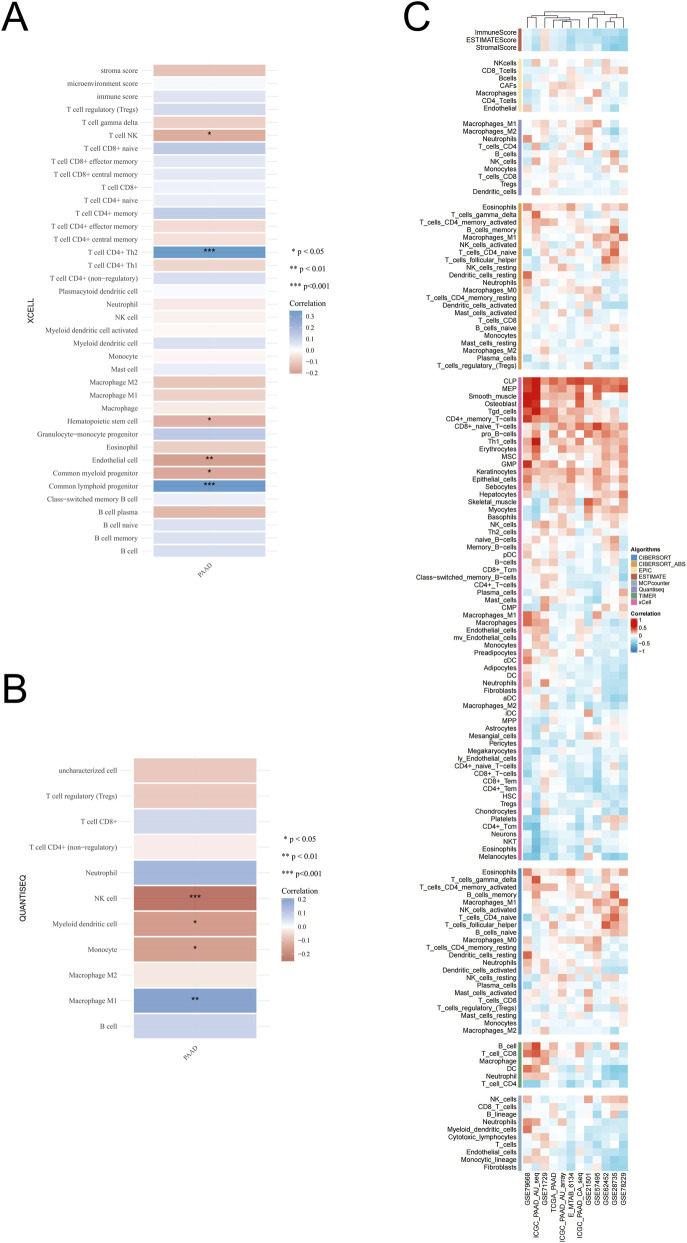
Correlation between EIF3H expression and tumor immune infiltration in PAAD. **(A)** Immune cell infiltration analysis using xCell algorithm showing correlation between EIF3H expression and various immune cell types. **(B)** Comprehensive immune infiltration analysis across multiple algorithms and datasets demonstrating consistent correlation patterns. **(C)** Immune cell correlation analysis using QUANTISEQ algorithm specifically highlighting significant immune cell subtypes. *P < 0.05, **P < 0.01, ***P < 0.001 Abbreviations: PAAD, pancreatic adenocarcinoma; NK, natural killer; Tregs, regulatory T cells; DC, dendritic cells; M1, macrophage type 1; M2, macrophage type 2; Th1, T helper 1 cells; Th2, T helper 2 cells; TEM, effector memory T cells; TCM, central memory T cells; CLP, common lymphoid progenitor; CMP, common myeloid progenitor; GMP, granulocyte-monocyte progenitor; HSC, hematopoietic stem cells; MPP, multipotent progenitor; MSC, mesenchymal stem cells; pDC, plasmacytoid dendritic cells; cDC, conventional dendritic cells; iDC, immature dendritic cells; aDC, activated dendritic cells.

Previous studies have confirmed EIF3H coordinates Hippo pathway-mediated tumorigenesis through catalytic control of YAP stability (Zhou et al., 2020). Our previous study demonstrated YAP1 was correlated with immune cell infiltration in pancreatic cancer (Sun et al., 2021). Therefore, we explored the relationship between EIF3H expression and immune cell infiltration in PAAD (Figure 10).

To explore the potential immunomodulatory role of EIF3H in PAAD, correlations between EIF3H mRNA expression and immune cell infiltration were assessed using TCGA-PAAD dataset. Multiple deconvolution algorithms, including ESTIMATE, TIMER, CIBERSORT, CIBERSORT-ABS, QUANTISEQ, MCPCOUNTER, XCELL, and EPIC, were employed to estimate immune cell abundances and their associations with EIF3H levels. The XCELL-based analysis revealed significant positive correlations between EIF3H expression and specific immune cell subsets such as CD4^+^ Th1 cells (*p* < 0.001) and common lymphoid progenitors (*p* < 0.01), while negative correlations were observed with B cell plasma (*p* < 0.05), endothelial cells (*p* < 0.05) and certain T cell subsets like CD4^+^ naive T cells (Figure 10A). Meanwhile, the QUANTISEQ algorithm demonstrated strong positive associations with M1 macrophages (*p* < 0.01), alongside negative correlations withmyeloid dendritic cells (*p* < 0.05), monocytes (*p* < 0.05) and NK cells (*p* < 0.001), suggesting EIF3H’s involvement in shaping a macrophage-dominant microenvironment (Figure 10B). To substantiate these findings, we employed a suite of immune infiltration analysis tools, including EPIC, ESTIMATE, TIMER, MCP-Counter, QuanTIseq, and XCELL, across 12 independent genomic datasets further highlighted consistent patterns across methods (Figure 10C). EIF3H exhibited robust positive correlations (r > 0.3) with M1 macrophages, neutrophils, fibroblasts, and endothelial cells in most algorithms (e.g., QUANTISEQ, XCELL, EPIC), while negative correlations (r < −0.3) were evident with CD8^+^ T cells, naive B cells, and plasma cells, particularly in TIMER and CIBERSORT. These findings indicated that elevated EIF3H levels fostered an immunosuppressive infiltrate enriched in myeloid cells and stromal elements, potentially contributing to immune evasion and tumor progression in PAAD.

### Analysis of immune regulatory genes, TMB, MSI, and immune checkpoints related to EIF3H in PAAD

**FIGURE 11:**
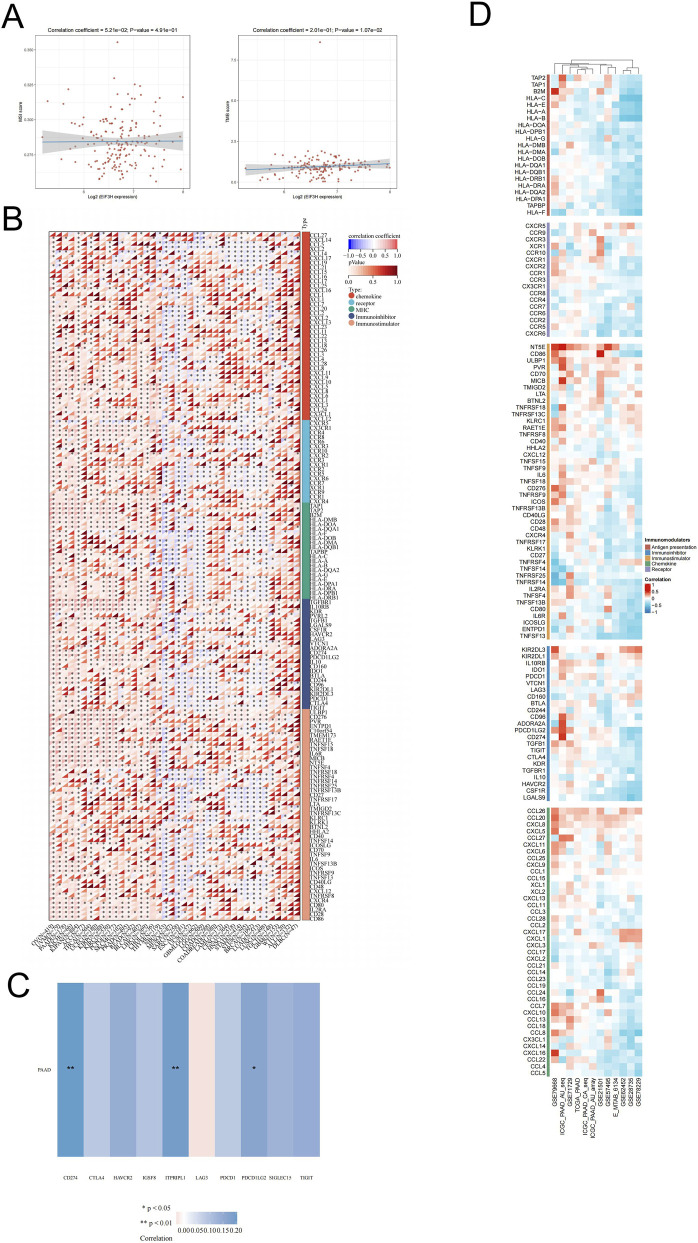
Integrated Analysis of Associations Between EIF3H Expression and Immune-Related Genes. **(A)** Associations of EIF3H expression with TMB and MSI in PAAD. **(B)** Pan-cancer associations between EIF3H expression and immune-related genes. **(C)** Analysis of the relationship between EIF3H expression and immune checkpoints in PAAD. **(D)** Associations between EIF3H expression and immune-related genes in PAAD across multiple immune infiltration tools and genomic datasets. *P < 0.05, **P < 0.01, ***P < 0.001. Abbreviations: PAAD, pancreatic adenocarcinoma.

To investigate the immunological role of EIF3H in PAAD, we analyzed its relationships with immune regulatory genes, TMB, MSI, and immune checkpoint molecules. Correlation analyses showed EIF3H expression exhibited a significant positive association with TMB (r = 0.201, p = 0.0107) but no significant correlation with MSI status (r = 0.0521, p = 0.491) (Figure 11A). Meanwhile, cluster analysis of immune regulatory genes across PAAD samples demonstrated coordinated expression patterns between EIF3H and multiple immunological factors, including HLA family members, antigen presentation-related molecules (e.g., TAP1), and other immune-modulating genes (Figure 11B). Immune checkpoint analysis revealed differential expression patterns of key molecules including CD274 (PD-L1), ITPRIPLI, and HAVCR2, with ITPRIPLI showing a significant positive correlation with EIF3H expression (Figure 11C). Furthermore, a comprehensive correlation heatmap across various cancer types (including PAAD) illustrated the interactions between EIF3H and immune-related genes (e.g., chemokines, receptors, MHC molecules, immunostimulators, and immunoinhibitors) (Figure 11D). In PAAD, EIF3H expression exhibited significant correlations with several immune genes. For instance, EIF3H was positively correlated with specific MHC-class I molecules and certain chemokine receptors. In terms of immune checkpoints, EIF3H expression in PAAD showed significant positive correlations with key checkpoint genes. Notably, CD274 (PD - L1, *P* < 0.01), PDCD1 (PD-1, *P* < 0.01), CTLA4 (*P* < 0.01), and HAVCR2 (TIM-3, *P* < 0.05) exhibited notable positive associations with EIF3H, as depicted by the correlation heatmap. These findings suggested that EIF3H may modulate immune responses in PAAD through interactions with immune regulatory networks, antigen presentation pathways, and immune checkpoint molecules, although no significant link with MSI was observed.

### EIF3H-associated immunotherapeutic responses and drug sensitivities in pancreatic adenocarcinoma

**FIGURE 12:**
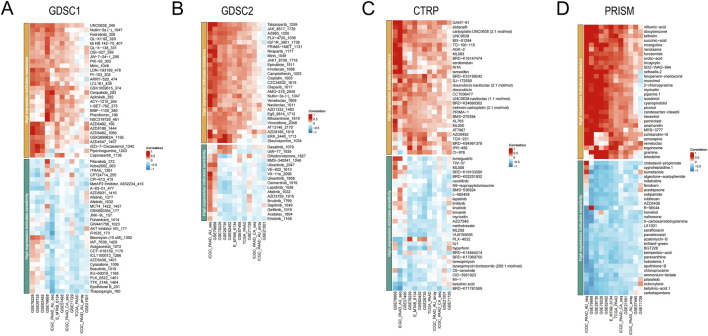
A comprehensive analysis of the correlation between EIF3H expression and drug response across multiple databases, along with its association with survival outcomes. **(A)** Correlation between EIF3H expression and drug resistance/sensitivity in the GDSC1 dataset. **(B)** Correlation between EIF3H expression and drug resistance/sensitivity in the GDSC2 database. **(C)** Correlation between EIF3H expression and drug resistance/sensitivity in CTRP the dataset. **(D)** Correlation between EIF3H expression and drug resistance/sensitivity in the PRISM dataset. *P < 0.05, **P < 0.01, ***P < 0.001. Abbreviations: CTRIP, Cancer Therapeutics Response Portal; PRISM, Preclinical Repurposing of Medicines; GDSC1/GDSC2, Genomics of Drug Sensitivity in Cancer 1/2; Log-rank, Log-rank test; Number at risk, Number of patients at risk at each time point.

To complement these immunotherapeutic insights, we examined EIF3H’s role in modulating drug sensitivities using comprehensive pharmacogenomic datasets, including GDSC1, GDSC2, CTRP, and PRISM, which encompass diverse therapeutic agents correlated with EIF3H expression levels (Figures 12A–D). Across these platforms, high EIF3H expression consistently predicted resistance to certain targeted therapies, such as MDM2 inhibitors (e.g., Nutlin-3a) and mTOR/PI3K pathway blockers (e.g., OSI-027, PKI-587), as indicated by positive Spearman correlations reflecting elevated IC50 values in high-expressing samples. This pattern implies that EIF3H-driven translational programs may sustain oncogenic signaling cascades, diminishing the efficacy of agents reliant on p53 activation or metabolic pathway inhibition. Conversely, negative correlations highlighted heightened sensitivities to proteasome inhibitors (e.g., Bortezomib), SERCA pump disruptors (e.g., Thapsigargin), and ferroptosis inducers (e.g., Erastin), suggesting that EIF3H upregulation exacerbates vulnerabilities in protein homeostasis and lipid peroxidation pathways, potentially through its deubiquitinating activity on key regulators like OGT. Extending these observations to additional datasets revealed similar trends, with high EIF3H levels conferring resistance to PARP inhibitors (e.g., Talazoparib), JAK inhibitors (e.g., JAK1-870), and anthracyclines (e.g., Epirubicin, Doxorubicin) in GDSC2 and CTRP cohorts, while enhancing responsiveness to multi-tyrosine kinase inhibitors (e.g., Sunitinib, Nilotinib) and BCL-2 antagonists (e.g., Venetoclax). In the PRISM dataset, correlations further underscored sensitivities to HDAC inhibitors (e.g., Panobinostat) and nucleoside analogs (e.g., Gemcitabine), aligning with EIF3H’s role in translational control that may amplify dependencies on epigenetic and nucleotide metabolism pathways. These differential sensitivities not only reflect EIF3H’s multifaceted impact on cellular resilience but also offer a framework for personalized therapeutic strategies, where EIF3H status could guide combinations of immunotherapies with sensitizing agents to overcome resistance and optimize outcomes in PAAD patients.

### PPI networks and functional annotations

**FIGURE 13:**
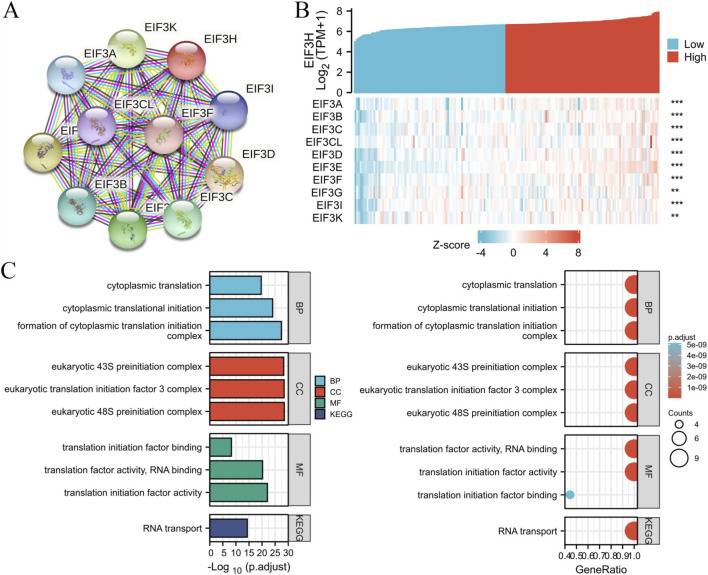
PPI networks and functional enrichment analyses of EIF3H. **(A)** A network of EIF3H and its co-expression genes. **(B)** Correlation analyses between the expression of EIF3H and co-expressed genes in PAAD. **(C)** Functional enrichment analyses of 10 involved genes. Abbreviations: BP: Biological process, CC: cellular component, MF: molecular function.

We conducted STRING database, GO, and KEGG analyses to construct PPI networks and functional annotations with EIF3H. Network of EIF3H and its 10 co-expression genes were showed in Figure 13A. Correlation analyses between EIF3H and co-expressed genes in PAAD from TCGA database were shown in Figure 13B. Functional annotations demonstrated that these genes were involved in eukaryotic 43S preinitiation complex, eukaryotic translation initiation factor 3 complex, eukaryotic 48S preinitiation complex (Figure 13C). Changes in the biological process (BP) and molecular function (MF) of EIF3H were correlated with cytoplasmic translation, cytoplasmic translational initiation, formation of cytoplasmic translation initiation complex, translation factor activity, RNA binding, translation initiation factor activity translation initiation factor binding (Figure 11C). KEGG molecular pathways was RNA transport (Figure 13C).

## Discussion

PAAD ranks among the most lethal human solid tumors, with high mortality driven mainly by challenges in early diagnosis and the limited efficacy of conventional therapies (Yamamoto et al., 2020; Knudsen et al., 2021). Current immunotherapeutic strategies for pancreatic cancer also remain constrained, yielding suboptimal clinical outcomes (Michl and Krug, 2018; Ma et al., 2020). EIF3H, a subunit of the EIF3, which is the largest and most complex of the initiation factors, has been implicated in oncogenesis (Cappuzzo et al., 2009). Overexpression of EIF3H has been shown to enhance cell proliferation, growth, and survival (Zhou et al., 2010). Elevated amplification and expression levels of EIF3H are observed in various cancers, including breast, prostate, liver, and lung adenocarcinoma (Hu et al., 2020; Mahmood et al., 2014; Zheng et al., 2021). Mechanistic studies have revealed that high EIF3H expression inhibits Myc-dependent apoptosis in primary prostate cells and promotes protein translation via cooperation with MYC(27, 33). Furthermore, the overexpression of EIF3H has been found to induce malignant transformation in immortalized NIH-3T3 cells within breast, prostate, and liver cancers (Savinainen et al., 2006). Recent work further identifies EIF3H as a critical translational initiation factor that modulates the Hippo-YAP pathway, a key regulator of tumorigenesis across several cancer types (Zhou et al., 2020), (Sun et al., 2016; Yuan et al., 2018). In our prior study, we demonstrated that YAP1 correlates with patient outcomes and tumor immune cell infiltration in pancreatic cancer (Sun et al., 2021). Given the limited understanding of EIF3H’s molecular mechanisms in tumors, this research focuses on elucidating the role and regulatory mechanisms of EIF3H in PAAD.

Our comprehensive study identified EIF3H as a crucial oncoprotein in PAAD, acting via an integrated regulatory axis bridging non-coding RNA networks, translational control and immune microenvironment remodeling. PAAD’s consistently dismal prognosis underscores the urgent need for novel molecular drivers targeting its inherent aggressiveness and profound immunosuppression. EIF3H’s widespread overexpression across various cancers places it within a conserved oncogenic program, and its marked upregulation in PAAD, validated across independent cohorts and at the protein level, indicates clinically relevant tissue-specific regulatory mechanisms. In PAAD, EIF3H expression was significantly associated with radiotherapy response, potentially due to its ability to promote radioresistance by enhancing the translation efficiency of key DDR mRNAs or cell cycle regulators (e.g., Cyclin D1, CDC25A), which enables PAAD cells to repair radiation-induced DNA damage more efficiently or re-enter the cell cycle rapidly. The correlation between elevated EIF3H expression and unfavorable prognosis, particularly in PAAD where it consistently predicted poorer overall, progression-free, and disease-specific survival across multiple datasets, strongly supported its potential as a reliable prognostic biomarker consistent with established observations that translation initiation factors frequently correlate with aggressive tumor phenotypes and adverse patient outcomes (Wu et al., 2025b; Ramalingam et al., 2023; Xiao et al., 2020). Furthermore, studies such as Zhang et al. (2025b) highlighted the importance of advance care planning in improving outcomes for advanced cancer patients, underscoring the clinical value of integrating prognostic biomarkers like EIF3H into optimized care strategies (Zhang et al., 2025b). Additionally, Li et al. (2014) described the clinical characteristics and management of acute pancreatitis, emphasizing vigilance regarding drug-induced pancreatitis, which complemented the translational relevance of EIF3H in pancreatic adenocarcinoma (Li et al., 2025).

Elucidation of the AC005034.3/hsa-miR-126-5p/EIF3H regulatory axis represents a key advance in understanding post-transcriptional regulation of oncogenic translation in PAAD. While EIF3H’s pivotal role in translation initiation is well-established across contexts, our identification of this specific ceRNA network clarifies how non-coding RNAs modulate EIF3H expression in PAAD. The well-recognized tumor suppressor function of miR-126-5p across cancers supports its inhibitory role in this axis, and the characterization of AC005034.3 as the functional lncRNA component adds a novel layer to the intricate regulatory network governing EIF3H expression. This axis holds clear clinical significance, as all three components show consistent prognostic implications and the inverse correlation between miR-126-5p and both EIF3H and AC005034.3 validates the ceRNA hypothesis. Comprehensive validation via correlation, expression and survival analyses provides robust evidence for the biological relevance of this regulatory circuit, rather than a mere statistical association.

EIF3H’s immunomodulatory function in the PAAD tumor microenvironment is a critical component of its oncogenic activity. Multi-algorithm deconvolution analyses consistently showed that EIF3H-high tumors exhibit an immunosuppressive milieu, with abundant M1 macrophages and neutrophils and reduced cytotoxic T cells and plasma cells. This distinct immune profile, validated across twelve independent datasets and eight computational methods, demonstrates that EIF3H not only drives intrinsic oncogenic pathways but also actively shapes an immune-evasive tumor environment. Strong correlations with immune checkpoint molecules including PD-L1, PD-1, CTLA-4 and TIM-3 further support this immunomodulatory role and imply relevance for immunotherapy responsiveness. Additionally, EIF3H’s positive correlation with tumor mutational burden suggests it may regulate neoantigen production, while the lack of association with MSI indicates this process relies on alternative pathways. The focus on EIF3H’s involvement in immune modulation, particularly its positive correlations with immune checkpoint molecules, illuminated a potential mechanism for immune evasion, underscored its relevance as a target for combined immunotherapies, and significantly enhanced our understanding of translational control in cancer—extending beyond cell-autonomous functions to encompass broader immunological interactions (Yan et al., 2025; Pan et al., 2025). The detailed analysis of EIF3H’s role in modulating the tumor microenvironment, particularly its association with immunosuppressive cell populations, illustrated the importance of integrating molecular and immune profiling to understand tumor progression, complementing our previous findings that illuminated a potential immune evasion mechanism, underscored EIF3H’s relevance as a combined immunotherapy target, and significantly enhanced our understanding of translational control in cancer—extending beyond cell-autonomous functions to encompass broader immunological interactions (Duan et al., 2024; Luo et al., 2024).

Within the context of the current therapeutic landscape for PAAD, our findings on drug sensitivity provided clinically actionable insights. The consistent resistance to MDM2 inhibitors and mTOR/PI3K pathway blockers observed in high-EIF3H expression models, together with the correlation between elevated EIF3H levels and resistance to specific targeted therapies, suggested that EIF3H-driven translational programs sustained oncogenic signaling through alternative pathways (Molnár et al., 2024). This finding might have accounted for the limited efficacy of these agents in clinical trials, thereby supporting the rationale for therapeutic strategies targeting EIF3H. In contrast, the increased sensitivity to proteasome inhibitors, ferroptosis inducers, and certain targeted agents highlighted therapeutic vulnerabilities that could be leveraged in clinical settings. The drug sensitivity profiling, which identified resistance to mTOR pathway inhibitors and sensitivity to proteasome inhibitors, extended mechanistic insights and offered valuable guidance for personalized therapeutic strategies. These patterns were consistent with EIF3H’s involvement in protein homeostasis and implied that pharmacological targeting of these pathways could be particularly effective in tumors with high EIF3H expression. The association with gemcitabine sensitivity was particularly significant, given its status as a first-line therapy in PAAD, and might offer a stratification strategy for treatment selection.

Among translation initiation factors analyzed in PAAD, EIF3H uniquely associated extensively with immune regulation and non-coding RNA networks. While EIF3A and EIF3B are implicated in multiple cancers, their mechanisms are largely restricted to direct oncoprotein translational control. Our prior identification of EIF3H’s link to YAP stability further integrates it into the Hippo pathway, supporting its role as a convergence point for oncogenic signaling. Functional annotation analyses confirmed EIF3H’s central role in translation initiation complexes, providing mechanistic support for its broad oncogenic effects, and specific RNA transport pathway identification revealed new avenues to explore its mode of action.

Despite robust support for the AC005034.3/hsa-miR-126-5p/EIF3H axis from our bioinformatics and expression analyses, this study has several limitations. First, direct molecular interactions and causal relationships within the proposed axis remain unconfirmed, as luciferase reporter assays and gain- or loss-of-function experiments were not performed. Second, clinical validation was limited to 12 paired samples due to resource constraints, potentially restricting the generalizability of our findings; larger multi-center cohorts with long-term follow-up data will be critical for future studies to enhance clinical relevance. Third, while our correlative analyses suggested EIF3H contributes to immunosuppression, mechanistic insights into its regulation of immune cell function lack experimental support, and co-culture systems plus *in vivo* models are planned to address this gap. Fourth, drug sensitivity predictions were purely computational, and wet-lab validations using EIF3H-manipulated cell lines are needed to verify these potential therapeutic vulnerabilities. Moving forward, future research will prioritize developing direct EIF3H inhibitors and exploring combination strategies—particularly with immunotherapies—to overcome the immunosuppressive microenvironment of EIF3H-high tumors.

## Conclusion

**FIGURE 14:**
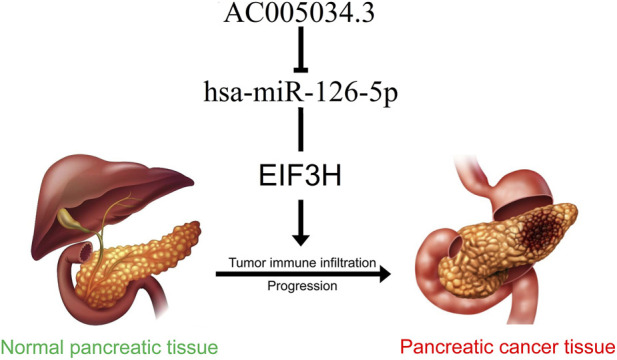
The model of AC005034.3/hsa-miR-126-5p/EIF3H axis in carcinogenesis of PAAD.

In conclusion, this study redefined EIF3H from a mere translation factor to a multifunctional oncoprotein that orchestrated tumor growth, metastatic progression, and immune evasion in PAAD via the novel AC005034.3/hsa-miR-126-5p regulatory axis (Figure 14). The identified upstream ncRNA pathway, particularly the AC005034.3/hsa-miR-126-5p/EIF3H axis, opened avenues for novel therapeutic interventions targeting non-coding RNAs, consistent with current research emphasizing ncRNA-mediated regulation of oncogenic pathways (Zeng et al., 2022; Wu et al., 2024). The extensive scope of our analysis, encompassing pan-cancer expression profiles, prognostic significance, regulatory mechanisms, interactions within the immune microenvironment, and therapeutic implications, established a solid foundation for future translational research. These findings not only enhanced our fundamental understanding of pancreatic cancer biology but also presented immediate clinical opportunities for prognostic stratification and therapeutic intervention.

## Data Availability

The original contributions presented in the study are included in the article/Supplementary Material, further inquiries can be directed to the corresponding authors.

## Glossary

EIF3H: eukaryotic translation initiation factor 3 subunit H
TCGA: the Cancer Genome Atlas
ncRNAs: noncoding RNAs
PPI: Protein–Protein Interaction
EIF3: eukaryotic translation initiation factor 3
YAP1: Yes-associated protein-1
GTEX: Genotype-Tissue Expression
miRNAs: microRNAs
lncRNAs: long noncoding RNAs
CI: confidence interval
TIMER: Tumor Immune Estimation Resource
GEPIA: Gene Expression Profiling Interactive Analysis
KM plotter: Kaplan-Meier plotter
ENCORI: Encyclopedia of RNA Interactomes
GO: Gene ontology
KEGG: Kyoto Encyclopedia of Genes and Genomes
FC: fold-change
HNSC: head and neck squamous cell carcinoma
KIRC: kidney renal clear cell carcinoma
RNA Seq V2 RSEM: mRNA expression z-scores
RPPA: protein expression Z-scores
CNA: copy-number alterations
ECL: extracellular loop
EDG: endothelial differentiation gene
GEO: Gene Expression Omnibus
GEPIA 2: Gene Expression Profiling Interactive Analysis 2
GPCRs: G-protein coupled receptors
HR: Hazard ratio
BLCA: bladder urothelial carcinoma
BRCA: breast invasive carcinoma
KICH: kidney chromophobe
KIRP: kidney renal papillary cell carcinoma
LIHC: liver hepatocellular carcinoma
ESCA: Esophageal carcinoma
LUAD: lung adenocarcinoma
LUSC: lung squamous cell carcinoma
PRAD: prostate adenocarcinoma
READ: Rectum adenocarcinoma
UCEC: uterine corpus endometrial carcinoma
CHOL: cholangial carcinoma
COAD: colon adenocarcinoma
CECS: Cervical squamous cell carcinoma and endocervical adenocarcinoma
STAD: stomach adenocarcinoma
DLBC: Lymphoid Neoplasm Diffuse Large B-cell Lymphoma
THCA: Thyroid carcinoma
GBM: Glioblastoma multiforme
PAAD: Pancreatic adenocarcinoma
THYM: Thymoma
ACC: Adrenocortical carcinoma
BLCA: bladder urothelial carcinoma
PCPG: Pheochromocytoma and Paraganglioma
UCEC: uterine corpus endometrial carcinoma
UCS: Uterine Carcinosarcoma
OS: overall survival
DFS: disease-free survival
RFS: relapse-free survival
PPS: post-progression survival
DSS: disease-specific survival
DMFS: distant metastasis-free survival
PFI: Progress free interval
FP: first progression
TAM: Tumor-associated macrophages
NK cells: natural killer cells
MDCs: myeloid dendritic cells
Tfh: follicular helper T cell
Th: T helper cell
CircRNAs: circular RNAs
BP: biological process
MF: molecular function
ceRNA: competitive endogenous RNA
CC: cellular component
