# Supplementary material for: AC005034.3/hsa-miR-126-5p/EIF3H axis: bioinformatics analysis, expression validation, and association with prognosis and immunosuppressive microenvironment in pancreatic adenocarcinoma
Source: Front Cell Dev Biol. 2026 Feb 25;14:1725060. doi: 10.3389/fcell.2026.1725060 (PMC12975753; doi:10.3389/fcell.2026.1725060)
Supplement: Supplementary file 1 [file Table1.docx]

Table S1 Clinical characteristics of patients with PAAD.

| Characteristics | overall |
| --- | --- |
| Age, n (%) |  |
| <= 65 | 94 (52.5%) |
| > 65 | 85 (47.5%) |
| Gender, n (%) |  |
| Female | 80 (44.7%) |
| Male | 99 (55.3%) |
| Histologic grade, n (%) |  |
| G1&G2 | 127 (71.8%) |
| G3&G4 | 50 (28.2%) |
| Pathologic stage, n (%) |  |
| Stage I&Stage II | 168 (95.5%) |
| Stage III&Stage IV | 8 (4.5%) |
| Primary therapy outcome, n (%) |  |
| CR&PR | 81 (57.9%) |
| PD&SD | 59 (42.1%) |
| Alcohol history, n (%) |  |
| No | 65 (38.9%) |
| Yes | 102 (61.1%) |
| Smoker, n (%) |  |
| No | 66 (45.5%) |
| Yes | 79 (54.5%) |
